# Performance and nutritional status of Holstein crossbred cows in a selected area of Bangladesh under the existing farming system

**DOI:** 10.5455/javar.2024.k818

**Published:** 2024-09-29

**Authors:** Md. Aliar Rahman, Rakhi Chowdhury, Khan Md. Shaiful Islam

**Affiliations:** Department of Animal Nutrition, Bangladesh Agricultural University, Mymensingh, Bangladesh

**Keywords:** Body weight, crossbred cows, milk yield, nutrition, profit

## Abstract

**Objectives:**

This study aimed to compare the body weight (BW), milk yield, nutritional status, and profitability of moderate genetic (MG) and high genetic (HG) merit of Holstein crossbred (HC) cows in a tropical region under the existing farming system.

**Materials and Methods:**

Data was gathered from 204 nursing cows of MG (*n = *99) and HG (*n = *105) merit of HC cows throughout a year in the dairy zone Keraniganj, Bangladesh. HC cows of MG and HG merit contained 50.0%–67.7% and 75.0%–87.5% Holstein blood, respectively. Data on genetic merit, BW, lactation stage and number, daily milk yield, feed intake, feed, and milk price were documented. All variables were except genetic merit analyzed using one-way analysis of variance.

**Results:**

HC cows of MG and HG merit had 433 and 493 kg BW (*p* < 0.01), and daily produced 11.99 and 14.06 kg milk (*p *= 0.07) with having 0.99 and 1.15 feed efficiency (*p *= 0.06), respectively but dry matter intake did not vary (*p* > 0.05). HC cows of both genetic merit daily offered surplus metabolizable energy and digestible crude protein through roughage and concentrate than their requirement (*p* > 0.05). The milk production cost of both genetic merit HC cows was alike (*p* > 0.05), whereas almost two times more profit was obtained in HG merit HC compared to MG merit HC cows (*p *< 0.05).

**Conclusion:**

HC cows of HG merit showed superior potentiality of milk yield, profit, and feed efficiency, whereas MG merit HC cows revealed inferior feed efficiency and milk yield.

## Introduction

The demand for milk and its products is expeditiously rising across the entire earth because of the massive population, health, and dietary awareness toward the consumption of nutritious diets [1]. By 2050, the world population will be around 9–10 billion and milk production will be amplified by almost 2.9% in tropical regions [2]. It assumes that around 61.0% of global milk will be produced in tropical countries by 2050 [3]. However, farmers in tropical countries used to herd Zebu-type indigenous cows (*Bos indicus*) which are bodily smaller, produce daily less milk over a shorter lactation period and are often fed inferior feed earlier in the day [4]. Despite these, tropical cows have superior genetic ability to adapt to seasonal changes, disease resistance, and tolerance towards tick infestation [5] but these are essential for temperate cows to adapt in the tropics. To overcome these problems and meet the enormous milk demand sustainably, high-yielding crossbred cows generally are propelled in tropical countries through artificial insemination of pure Holstein cows with tropical cows in intensive farming systems who can produce 23.2 kg of milk per day in temperate regions performing better feed conversion ratio [6]. The performance of temperate pure Holstein cows relies on proper nutrition, housing, heat stress mitigation systems, and decent husbandry practices [7]. So, it is essential to evaluate the performance of temperate breeds in tropical regions to hasten the milk yield in this area.

In order to combat heat stress and boost milk yield and immunity, crossbreeding between temperate and tropical breeds usually are practiced in tropical areas including South America, West Africa, and some parts of Southeast Asia in the early 19^th^ and 20^th^ centuries [7]. Temperate-tropical crossbreds typically perform more productively than tropical native breeds. It is observed that massively upgrading native breeds is a faster way to hasten milk yield [8]. The trend is also following in tropical countries and getting positive results at the farm level, and crossbred produce daily 08–20 kg milk with long lactation length and better feed conversion ratio [9]. However, cows of 87.5%, 75.0%, and 50.0% Holstein blood with 12.5%, 25.0%, and 50.0% tropical native cows daily yield 20.0, 15.2, and 18.0 kg of milk, respectively [10]. There is an inconsistent finding on which blood percentage of Holstein is suitable for tropical countries. In the tropics, these crossbred cows are fed haphazardly with low-grade roughage and concentrate without taking into consideration their body weight (BW), production, and stage of pregnancy. However, imbalanced nutrition is a main drawback to expressing the novel genetic potentiality of crossbred cows thus resulting in lower profit in dairy production [11]. So, this research was planned to assess and compare the milk yield, feed efficiency, nutritional status, profitability, and adaptability of different genetic merits of HC cows under the existing farming system.

## Materials and Methods

### Ethical approval

Animal handling and data collection were approved by the Animal Welfare and Experimentation Ethics Committee, Bangladesh Agricultural University, Bangladesh (AWEEC/BAU/2020(61)).

### Study area, farm selection, and dietary regime

As a tropical region, Keraniganj Upazila (Location: 23^o^70′06.5″ N, 90^o^39′73.0″E; an average temperature: 27.0°C ± 3.0°C, and humidity 70.0% ± 5.0%) in Dhaka, Bangladesh was selected to amass information on performance, nutritional status, and cost analysis of MG and HG merit HC nursing cows from June 2021 to July 2022. Among the three farms, total number of HC of MG and HG merits cows were 99 and 105, respectively. MG merit HC possessed 50.0%–67.5% Holstein blood and 50.0%–32.5% Sahiwal blood. Besides, HG merit HC contained 75.0%–87.5% Holstein blood and 25.0%–12.5% Sahiwal blood. In all farms, similar items concentrate and a variety of roughage including green grass, local grass, water hyacinth, and rice straw were given to nursing cows based on seasonal availability (Table 1). Cows of all three farms were given 7.5 mg fenbendazole/kg BW orally.

### Data collection, nutrient requirement calculation, and analysis

Each nursing cow‘s BW, parity number, stage of lactation, pregnancy status, and daily milk yield were all prudently recorded. At first, the BW of nursing cows was taken using Schaeffer’s formula [12], while the rest information was finally gathered from a specific cow’s record book. Each nursing cow was offered a variety of roughage and concentrate two times per day and noted precisely, and finally, the intake was calculated from the supplied and ort value. Each roughage and concentrate item were collected seasonally and the proximate components were measured according to Association of Official Analytical Chemists [13], while metabolizable energy (ME) was calculated by adopting the equation [14] and shown in Table 2. Using this nutritive value of these supplied roughage and concentrate feed items, dry matter (DM) intake, ME, and digestible crude protein (DCP) for maintenance and production for each nursing cow were calculated. Besides, ME and DCP requirement for the maintenance and production of each nursing cow was determined by adopting the formulae of Agricultural Research Council [15]. In addition, the sum of maintenance and production ME and DCP were denoted as total ME and DCP, respectively. Then, the balance for ME and DCP were calculated by subtracting the supplied from the requirements value of total ME and DCP. Furthermore, feed efficiency was calculated by dividing the daily milk yield by the daily DM intake. Then, the price per kg sold milk and the cost of feed (roughage and concentrate), feed additives, medication, vaccination, electricity, and artificial insemination were recorded.

**Table 1.** Adopted feeding system among three specialized dairy farms during the study period.

Feed items	Time frame	Farm 1			Farm 2			Farm 3	
		Feed	Amount (kg)		Feed	Amount (kg)		Feed	Amount (kg)
Roughage	January- July	Jumbo grass Local grass	15.0–20.0 1.0–2.0		Jumbo grass Molasses treated rice straw	13.0–18.0 1.5–2.5		Water hyacinth	25.0–30.0
	August- December	Water hyacinth Local grass	20.0–25.0 1.5–2.5		Local grass Molasses-treated rice straw	2.0-3.0 4.0–8.0		Water hyacinth	28.0–33.0
Concen-trate	January- July	Mixed bran Boiled concentrate mix	4.0–5.0 5.5–6.9		Mixed bran Compound feed	5.5–8.5 1.0–3.0		Mixed bran Compo-und feed	8.5–12.5 1.5–3.5
	August- December				Mixed bran Compound feed	6.5–10.5 1.9–3.5			

**Table 2.** Chemical composition of supplied feed items during the study period.

Supplied feed items	Proximate components (%)						ME* (MJ/kg DM)
	DM	CP	CF	EE	Ash	NFE	
Molasses-treated rice straw	88.12	5.17	34.89	1.52	10.06	48.36	8.51
Jumbo grass	15.01	9.50	32.80	1.80	10.70	45.20	8.79
Local grass	17.02	5.70	22.50	4.70	10.90	56.20	10.86
Water hyacinth	10.80	13.40	31.80	2.50	34.10	36.49	8.33
Mix bran	88.35	12.87	16.05	4.37	10.74	55.97	11.79
Boiled concentrate mix	27.58	10.13	6.81	4.05	9.12	69.89	13.34
Compound feed	88.09	22.08	6.20	5.28	10.95	55.49	13.43

*calculated value.DM: dry matter; CP: crude protein; CF: crude fiber; EE: ether extract; NFE: nitrogen free extract; ME: metabolizable energy

### Statistical analysis

All data were inserted in Excel, and DM, ME, and DCP supply and requirement for each nursing cow were calculated. Then, all data of MG and HG merit HC cows were analyzed in an independent sample *T*-test using IBM SPSS 2021 (Version 20.0; IBM Corp., Armonk, New York, USA), and the differences at *p* < 0.05 were reflected as statistically significant.

## Results

### Production performance

**Figure 1.**
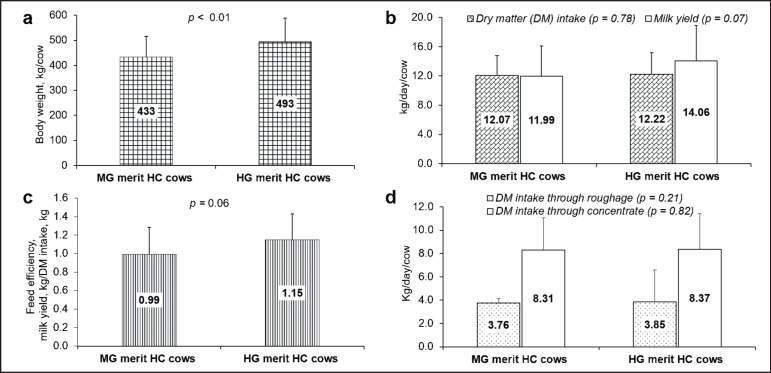
Influence of genetic merit on production performance of Holstein crossbred nursing cows. MG merit HC cows: moderate genetic merit Holstein crossbred cows; HG merit HC cows: high genetic merit Holstein crossbred cows; Kg: kilogram; p < 0.05: denotes statistically significant.

BW of MG and HG merit HC cows differed substantially, with the HG merit HC cows showing around 14.0% higher (*p* < 0.05; Fig. 1a), whereas total DM intake and DM consumption through roughage and concentrate did not vary (Fig. 1bd). However, the milk yield of HG merit HC cows was about 17.0% higher than that of MG merit HC (*p *= 0.07; Fig. 1b). Moreover, feed efficiency was obtained at 0.99 and 1.15 in MG and HG merit HC cows, respectively (*p* = 0.06; Fig. 1c).

### ME status

Compared to HC cows having MG merit, HG merit cows required about 12.0%, 9.0%, and 15.0% more total ME (*p *< 0.05), ME for maintenance (*p *< 0.05), and ME for production (*p* > 0.05), respectively (Table 3). But cows with HG merit offered 5.0% higher total ME and 7.0% ME via concentrate diet than cows with MG merit which was insignificant (*p* > 0.05). However, there was no difference in ME given by roughage and ME balance between MG and HG merit HC cows (*p *> 0.05).

**Table 3.** Influence of genetic merit on the metabolizable energy status of Holstein crossbred nursing cows.

Variables (MJ/cow/day)	MG merit HC cows	HG merit HC cows	SEM	*p*-value
**Supply**				
Total ME (Roughage + concentrate)	121.52 ± 31.41	128.14 ± 47.18	3.97	0.41
ME via roughage	27.73 ± 2.71	28.15 ± 2.15	0.24	0.38
ME via concentrate	93.79 ± 32.11	99.99 ± 48.14	4.05	0.45
**Requirements**				
Total ME (Maintenance+ production)	98.89 ± 23.59	110.97 ± 34.26	2.96	0.04
ME for maintenance	48.47 ± 9.87	52.91 ± 9.60	0.97	0.02
ME for milk production	50.42 ± 17.23	58.06 ± 28.15	2.34	0.10
**Balance**				
Total ME	22.63 ± 25.58	17.17 ± 46.34	3.72	0.47

MG merit HC cows: moderate genetic merit Holstein crossbred cows; HG merit HC cows: high genetic merit Holstein crossbred cows; ME: metabolizable energy; MJ: megajoule; SEM: standard error means; *p *< 0.05: denotes statistically significant.

**Table 4.** Influence of genetic merit on digestible crude protein status of Holstein crossbred nursing cows.

Variables (gm/cow/day)	MG merit HC cows	HG merit HC cows	SEM	*p*-value
**Supply**				
Total DCP (Roughage + concentrate)	861.54 ± 255.31	878.42 ± 278.06	26.12	0.83
DCP via roughage	176.08 ± 44.33	180.03 ± 41.64	4.19	0.64
DCP via concentrate	685.46 ± 230.02	693.01 ± 256.60	23.86	0.88
**Requirements**				
Total DCP (Maintenance + production)	676.91 ± 158.61	764.80 ± 225.10	19.67	0.03
DCP for maintenance	348.80 ± 77.80	383.51 ± 74.53	7.62	0.02
DCP for milk production	328.11 ± 107.53	381.29 ± 176.71	14.70	0.07
**Balance**				
Total DCP	184.63 ± 227.73	113.62 ± 216.67	21.98	0.08

MG merit HC cows: moderate genetic merit Holstein crossbred cows; HG merit HC cows: high genetic merit Holstein crossbred cows; DCP: digestible crude protein; gm: gram; SEM: standard error means; *p* < 0.05: denotes statistically significant.

### DCP status 

HC cows with HG merit sustainably required higher total DCP, and DCP for maintenance (*p* <0.05) and production (*p* = 0.07) by about 13.0%, 10.0%, and 16.0%, respectively, compared to cows with MG merit (Table 4). However, HC cows fed total DCP, and DCP via roughage and concentrate did not considerably differ between MG and HG merit of HC cows. Furthermore, the daily DCP balance for MG and HG merit of HC cows were inconsequential (*p* > 0.05).

### Cost analysis

Feed (roughage and concentrate), other, and total cost did not show significant variances between MG and HG merit HC cows, although HG merit HC cost somewhat more (*p* > 0.05; Table 5). However, income from sold milk revealed a significant trend, with HG merit HC cows receiving a 17.0% higher milk price than MG merit HC cows (*p *= 0.07). Moreover, HG merit HC cows daily generated almost two times higher profit than MG merit HC (*p *< 0.05).

## Discussion

## Production performance

Considerably higher BW (*p *< 0.01) but better daily milk yield (*p* = 0.07) and feed efficiency (*p* = 0.06) were perceived in cows of HG merit HC cows compared to MG merit HC, while the DM consumption through roughage and concentrate did not show any variation between both genetic merits. Notably, genetic merit and BW are positively correlated and both of these traits are allied to milk yield [10,16]. In the present study, HG potentiality showed greater BW, better milk yield, and feed efficiency and MG showed lower value which supports the previous findings. However, cows of 52.0% and 92.0% Holstein blood substantially influence daily milk yield (35.9 *vs.* 39.6 kg) but have no impact on BW (603 *vs.* 601 kg), total DM intake (17.9 *vs.* 18.2 kg) and feed efficiency (0.51 *vs.* 0.53) in temperate region, respectively [17]. However, in current research, it was observed a lower and significant variation between BW (433 *vs.* 493 kg), but not on milk production (11.99 *vs.* 14.06) and total DM intake (12.07 *vs.* 12.22 kg) and feed efficiency (0.99 *vs.* 1.15) of MG and HG merit HC cows, respectively. BW of 52.0% and 92.0% HC cows do not influence due to both cows containing 42.0% and 8.0% Friesian blood, respectively [17]. However, a lower and significant variation in BW of MG and HG merit HC cows was obtained due to both cows containing the blood of Sahiwal at 25.0% and 12.5%, respectively. Due to the presence of a higher percent of Holstein merit, cows showed the tendency of better milk yield and feed efficiency, without affecting the total DM intake [10,17]. Another crucial factor of lower BW and milk production of HC cows is the environmental factors especially temperature since genetic potential relies on environment, nutrition, and their association [18,19]. Improper nutrition especially low-quality rice straw might be another reason for lower BW and milk yield of MG and HG merit HC cows in this study, since poor nutrition to heifers causes a lower growth rate and reduces the expression of optimum genetic potentiality i.e., milk production [20]. Besides, it was illustrated that daily milk production of MG and HG merit HC cows differ from 13.8 to 14.1 kg in Thailand [21] which supports our findings. Cummins [22], illustrated that increasing the genetic merit of Holstein cows improves milk production which is in line with our findings. However, the milk yield of Holstein and Sahiwal crossbred cows is about 6.0 kg under village conditions [23] and 7.9–9.2 kg under intensive farming systems [24] which are lower and inconsistent with the findings of the present study. Since in the previous study [23,24] crossbred cows are given a straw-based diet and have lower genetic merit, respectively. In the current study, the crossbred cows of both genetic merit produced lower milk compared to cows of temperate countries. The environmental temperature, and proper nutrition (green grass-based) and their interaction might be another reason for temperate countries to produce more milk compared to our study [22].

**Table 5.** Influence of genetic merit on cost analysis of Holstein crossbred nursing cows.

Variables (Dollar^#^/cow/day)	MG merit HC cows	HG merit HC cows	SEM	*p*-value
A. Total feed cost (Roughage +concentrate)	4.70 ± 1.18	4.78 ± 1.28	0.12	0.73
i. Feed cost for concentrate	3.63 ± 1.20	3.68 ± 1.32	0.12	0.82
ii. Feed cost for roughage	1.07 ± 0.11	1.10 ± 0.11	0.01	0.21
B. Other cost	2.11 ± 0.53	2.15 ± 0.58	0.05	0.73
i. Total production cost (A+B)	6.81 ± 1.71	6.93 ± 1.86	0.18	0.73
ii. Price of sold milk (Income)	8.14 ± 2.79	9.55 ± 4.63	0.38	0.07
iii. Profit (II-I) over feed and other cost	1.33 ± 2.16	2.62 ± 3.61	0.30	0.03

MG merit HC cows: moderate genetic merit Holstein crossbred cows; HG merit HC cows: high genetic merit Holstein crossbred cows; Other cost: labor, feed additive, medication, vaccination, electricity, artificial insemination; SEM: standard error means; *p *< 0.05: denotes statistically significant; ^# ^1 Dollar: 97.0 Bangladeshi Taka.

## Nutritional status

The ME and DCP requirements for milk production of MG and HG merit HC cows did not show variation, whereas the ME and DCP requirements for maintenance showed significant differences, and greater value was obtained in HG merit HC cows. Noticeably, greater BW of HG merit HC cows results in more ME and DCP requirements for body maintenance and vice versa [20]. However, ME and DCP requirements for milk production of MG and HG merit HC cows obtained statistically alike, but the higher HG merit HC cows is due to better milk yield [20]. Consequently, in this study total calculated ME (99 *vs.* 111 MJ/day) and DCP (677 *vs.* 765 gm/day) for maintenance and milk production requirements showed substantial variation between the genetic merit of HC cows, and higher value was obtained in HG merit HC cows, while crossbred cows having a BW of 400–500 kg and producing daily 10 kg milk result in a substantial variation for ME (98–103 MJ/day) and DCP (790–800 gm/day) requirement for maintenance and production [25]. Though cows of both HC showed significant variation for total ME and DCP requirement, they were given insignificant and comparable ME and DCP through roughage and concentrates. After body maintenance and milk production, HC cows of both genetic merit had daily around 23.0 and 17.0 MJ ME for maintaining pregnancy and weight gain respectively, but crossbred cows having nearly 500 kg BW requires daily around 25 MJ ME in the third trimester [25], almost daily requires 10–15 MJ ME for pregnancy [26]. Besides, HC cows with MG and HG merit were given daily excess DCP (185 *vs.* 114 gm) after meeting body maintenance and production requirements, whereas crossbred cows daily require around 76.2–86.6 gm DCP [25] or 83.6–97.4 gm DCP [27] for pregnancy maintenance. Cows of MG merit HC daily were offered around two times higher DCP than their requirement and compared to HG merit HC cows. In this study, it seems that HC cows of both genetic merit were given an almost similar and higher amount of ME and DCP than their requirements, respectively.

In the current research the DM, ME for maintenance, and total ME requirements and intake of lactating cows differed significantly among the farms of two genetic merit, this significant results are mainly due to the difference in the BW and milk production status of dairy cows among the farms and two genetic merit of HC cows [14,20].

## Cost analysis

In the current research, profit was obtained in dairy farming which supports the results of [28]. To our knowledge, no research has been performed on economic analyses between MG and HG merit HC cows in tropical countries, but higher profit was obtained in HG merit HC cows compared to MG merit HC cows, though all variables were insignificant in this study. This variation may be due to inappropriate feeding practices without considering genetic potentiality and higher milk production of HG merit HC dairy cows [29,30].

## Conclusion

Under the existing farming system, Holstein crossbred cows of high genetic merit had better BW, feed efficiency, milk yield, nutritional status, and profitability compared to moderate genetic merit. However, the performances of both genetic merit are inferior compared to temperate animals. This means that crossbred cows with both genetic merit had the genetic potential to produce more milk and be more profitable if they were given balanced nutrients and a better farming system. So, intensive research is a prerequisite to determine the real production performance of crossbred cows feeding optimum quality ration in accordance with the different levels of nutrients.

## List of Abbreviations

ARC, Agricultural Research Council; BW, body weight; DCP, digestible crude protein; DM, dry matter; gm, grams; h, hours; HC, Holstein crossbred; HG, high genetic; Kg, kilograms; ME, metabolizable energy; MG, moderate genetic; mg, milligram; MJ, megajoule.
